# The Care and Management of the Insane in Texas

**Published:** 1888-08

**Authors:** A. N. Denton

**Affiliations:** Late Superintendent State Lunatic Asylum, Austin, Texas


DANIEL'S

### Texas Medical Journal.

A Representative Organ of the Medical Profession, and an Exponent of Rational Medicine; devoted to the Organization, Advancement and Elevation of the Profession in Texas.

PUBLISHED MONTHLY.—SUBSCRIPTION $2.00 A YEAR.

Vol. IV. AUSTIN, AUGUST, 1888. No. 2.

### ORIGINAL ARTICLES.

CONTRIBUTED EXCLUSIVELY TO THIS JOURNAL.

*The Articles in this Department are accepted and published with the understanding that we are not responsible for, nor do we indorse the views and opinions of the writers by so doing.*

### THE CARE AND MANAGEMENT OF THE INSANE IN TEXAS.

BY A. N. DENTON, M. D.,

*Late Superintendent State Lunatic Asylum, Austin, Texas.*

Read at Austin District Medical Society, June 14, 1888.

IN a paper read before this body on the 8th of last March, I discussed briefly the care and management of the insane in Texas. As indicated by the title of the paper, I endeavored to show, on that occasion, that the management of this unfortunate class of our defective population in Texas, was peculiar, in the fact that the government of the asylums was changed, or you may say revolutionized, with the advent of each incoming governor; that there was not only a change of the superintendents, but frequently the entire working force of the asylums was changed, leaving the institutions in the hands of persons entirely without experience either in the care and management, or treatment of the insane.

In continuing the subject, it is proper to add that my own want of experience in management when I was placed in charge of the State Institution near this city, and the difficulties which I then encountered, deeply impressed me with the importance of previous preparation and experience before assuming the burden of such great responsibilities and exalted trusts.

Much more might be said on the great importance of experience in the management and treatment of the insane, and almost unlimited quotations might be made from the greatest authors and alienists, as to its usefulness, but limited time and space forbid.

In further pursuing the subject in this paper, I shall offer some additional thoughts on the subject of management, leaving the subject of the causes, pathology and treatment of the insane to be discussed in a paper, or papers, which I purpose reading before this society at some future time.

In the experience of the writer in the management of the insane, no principle or fact was more deeply impressed upon his mind than the necessity for a universal law of kindness in the management. This law should pervade every department of the institution, and every patient of every grade and color should be able to feel and realize its benign influence. It is not an easy task to always enforce this heavenly law.

Persons without experience would naturally suppose that human sympathy would incline everyone to be kind and sympathetic to the most deeply afflicted class of the human race ; and in exceptional cases this is true, but unfortunately in a majority of instances it is not the case. Abundant observation teaches that the great majority of men and women who apply for employment in asylums, are totally unfit for the care of the insane. But few have the necessary intelligence and breadth of mind to appreciate the responsibilities of the trust, and hence, when assailed by the patients, either with abusive language or physical force, they are unable to suppress an impulse of resentment, and such feelings are often entertained far beyond the period of the incident which gave them birth. Prejudice is thus established in the breast of the person whose duty it is to tenderly care for the insane, and too often abuse and ill treatment of the patient is the result. In many instances, unless the patient is intimidated, such abuses and ill treatment is reported by the patients to the superintendent, and in a very large percentage of such cases, when investigation is made, a positive conflict of testimony arises between the patient and employe, and unless evidences of physical violence is found upon the person of the patient, it is frequently found to be impossible to form a satisfactory conclusion as to the guilt or innocence of the accused.

Such difficulties are almost constantly arising in all large institutions for the care of the insane, where sufficient care is not exercised in the selection of employes, and even where the utmost vigilence is used, it seems almost impossible to prevent bad men and women from occasionally creeping into the service of such institutions. Unfortunately many persons will present themselves for employment, bearing the best recommendations, who are unworthy of trust. There is a class of persons, who travel from one asylum to another, and from one State to another, frequently, with excellent recommendations, who should be especially avoided. They are generally mischief-makers and unworthy of trust. In the selection of attendants and other employes, much, of course, will depend upon the judgment and shrewdness of the superintendent; and even with good judgment, constant vigilance and honest intentions, he will inevitably be the occasional victim of his own mistakes in the selection of employes. The difficulties encountered in the selection and retention of honest and faithful employes have, in many of the asylums, been productive of almost endless confusion and trouble in the management.

These difficulties have suggested the advisability of the establishment of training schools for nurses or attendants. A few of such institutions have already been established. While the inaugurators of these institutions claim for them success, and that the graduates from such schools show an aptitude and faithfulness not possessed by the untrained, it is, perhaps, too early to predict the ultimate result of these enterprises. To the writer, however, it seems a step in the right direction, and entertaining this opinion, he was already proposing to establish such a school in connection with the asylum near the city at the time of his retirement from the superintendency.

The average attendant under existing conditions and methods is apt, unless carefully instructed, to fall into the error, that he is employed to manage and control, instead of to nurse, and tenderly care for the patients in his charge. He easily falls into the error that order and neatness in the ward is the only requisite ; and unfortunately the inexperienced superintendent falls into the same error, and thus the comfort and happiness of the patients are often sacrificed to the accomplishment of this end. The importance of order and system in every department of such institutions, and also the continual maintainance of the best sanitary condition, is not underestimated by the writer, but the enforcement of such military precision in the personal conduct of the patients, as the writer has observed in a few of the asylums of the country, is the refinement of cruelty.

Attendants are held responsible for the condition of their wards, and in order that they may always be kept neat and orderly, with the least exertion on the part of the attendants, the patients are forced to occupy their rooms a great portion of the day, or if allowed in the halls, are forced to keep their seats, when they should be allowed to exercise in the hall. In other instances, if the unfortunate patient is restless and noisy, the attendant will insist on the privilege of controlling the movements of such patient by the use of mechanical restraints. To prevent the unnecessary and cruel employment of mechanical restraint, is one of the most diffiucult tasks of a conscientious superintendent. In order to succeed he must adopt and enforce the most exact rules.

The only justification for the use of mechanical restraints were enumerated in a former communication to this Society, and therefore need not be repeated.

From careful and extended observation, the writer has formed the opinion that with the average population of the asylums of this country, the restraints should never exceed a maximum of one per cent. of the entire population. This limit was rarely, if ever, exceeded during the last three years of the writer's administration of the affairs of the asylum near this city, and there were often periods of days during which not a single patient was restrained. These facts are evidence sufficient as to the practicability of abolishing restraints altogether in the management of the insane. But it should not be done merely for the name of having done so, and it is difficult to understand how it can always be done with justice to the patients. Probably the most useful of all adjuncts in the treatment and management of the insane, is employment. While much careful study and experiment has been bestowed upon this subject, especially in the northern States, in the opinion of the writer, its importance has not yet been fully appreciated. Idleness is, in many instances, the indirect, and in some cases, the direct cause of insanity. Its continuance during asylum life is detrimental, in every way, to the interest of the patient. Of course allusion is only made to that portion of the population whose physical and mental condition is such as to warrant them in engaging in some kind of labor. In order to the maintenance of physical and mental vigor, the constitution of man demands employment. It is an absolute essential to his health and happiness, and without it bodily deterioration and mental decay are inevitable. If he has nothing on which to bestow the labor of his body and mind, the former will soon become diseased, and the latter will share in the destructive process of gradual decay and death. An asylum for the care of the insane, without provisions for the employment of its inmates, is, at best, but a dreary prison, inferior to work-houses or penitentiaries, with respect to the contentment and happiness of its inmates. It is difficult, without experience, to realize the dreary wretchedness of a prison life without employment. Such a fate is, perhaps, the saddest allotted to man, and can only be productive of evil to the unhappy victim.

Providing employment for all the inmates capable of performing labor, of a large institution for the care of the insane, is a problem not easily solved; but its accomplishment is possible, and the benefits to be derived will more than compensate for the extraordinary effort. In this country, where land is cheap, agriculture, and kindred occupations, offer the best field for the employment of the male population. All, of course, could not be employed in this way, but a small proportion could be employed in mechanical occupations. The systematic employment of females presents greater difficulties; but observation and experiment have convinced the writer that even these difficulties are not insurmountable.

As above indicated, the chief object for procuring employment for every patient capable of performing labor of any kind, is the physical and mental improvement of the patient. In view, however, of the grave financial problem involved in the governmental support of the ever increasing proportion of the defective population of the country, it is certainly a matter of the greatest importance to render institutions for the care of the insane as nearly self-supporting as possible.

It is the opinion of the writer, based upon extended observation and experiment, that as large a proportion as sixty per cent. of the average asylum population of this country can be induced to engage in some kind of remunerative employment, which, if properly systematized, would not only add to the per cent. of restorations, but would materially add to the revenue of the institution. The possibilities in this direction have not yet been fully tested, but there is scarcely a doubt but at least twenty-five per cent. of the entire expense for support might be thus saved, and that with great benefit to the inmates. Of course, it is not intended in the above remarks to convey the idea that employment is ever to be compulsory; it is never necessary. Example and persuasion are sufficient to induce all, or nearly all, to engage in some kind of labor who are capable of performing it.

Everything in this direction, will depend upon the ability and tact of the Superintendent. His personal supervision and active interest in the details of every department, is essential.

This last observation leads to the consideration of the subject of proper asylum capacity, under the same administration or government.

Much has been written upon the subject by alienists of larger experience and geater ability than the writer, and I need not detain you with a recital of the details of the discussion. It seems that the conclusion arrived at, is, that the average number of insane under one superintendency, or government, should not exceed from three to four hundred. In the opinion of the writer, if the interests and well being of the insane are alone considered, the verdict is just and true. If, however, the question of economy of support is to be considered, without reference to the employment of the inmates, the number above mentioned might be quadrupled with advantage. But if such a large number is placed under one management, the superintendent can only act as the executive head and medical director of the institution, the executions of the details in most of the departments being delegated to his subordinates. As above remarked, where economy of support is the chief consideration, such aggregations are preferable, but they are objectionable on account of the impossibility of scrutiny of details of management and treatment by the responsible head of the institution. The superintendent is generally a bonded officer, the only one connected with the institution.

To place him in charge of such numbers of insane as renders it impossible for him to personally observe the details of management and treatment, and at the same time hold him responsible for all the abuses and neglect of his subordinates, would be a manifest injustice. If the government of the asylum is single, with one responsible executive head, the number under his care should not be so great as to render it impracticable for the superintendent to see all of his patients each day, and to cerefully observe and scrutinize the details of management and treatment in every department; and notwithstanding the opinion and practice of other gentlemen in the specialty, for whose opinion the writer has profound respect, he is thoroughly convinced from careful observation and experience, that such scrutiny on the part of the superintendent is indispensable to the well being of the inmates. Where the government is dual as to superintendent, with one board of directors, the number of patients may, of course, be increased. Such governments, however, have not given satisfaction to the experimentors, and are, therefore, not to be commended.

The safest and best governments for such institutions is that in which the entire responsibility for the internal management is lodged in one individual, whether he is designated as superintendent, or medical director, or by any other title. In addition, there should be a board of directors, whose duties should be advisory and supervisory with power to remove the superintendent for cause, and appoint his successor.

I have hitherto said nothing on the subject of classification, which simply means the segregation of the different types of insanity. In public institutions (which are only considered in this paper), perfect classification is generally impracticable and undesirable.

Appropriations by the Legislature are usually made for about one attendant to 12 to 18 patients. With such a meagre force, it is scarcely possible to care for every class separately, but by a judicious mixing, or sandwiching, so to speak, of a few acute and troublesome cases, with the quiet or convalescent ones, much good may be accomplished with a smaller force of attendants.

Epileptics, on account of the horrible nature of the convulsions to which they are subject, and the terror and excitement which they produce in the minds of others, should, if possible, be cared for in a separate ward, or better still, in an asylum conducted especially for their benefit. But, by far the most important feature of classification is the segregation of the sick and bedridden from those who are in the enjoyment of some degree of physical health. No public institution of this character should be without a hospital or infirmary, where the sick may be properly cared for.

To the writer, who some years ago, visited more than twenty of the larger public Insane Asylums, of the United States, nothing excited so much surprise as the general absence of hospitals in connection with these institutions, for the separate care of the sick and bedridden. A few were provided with this necessary adjunct, but the great majority were without them. The hospital should be in a separate building, with male and female departments, and at such distance from the other building of the institution as to escape most of the noises incident thereto. The capacity of the hospital should be sufficent to accommodate about five per cent. of the maximum population of the asylum. It should be so constructed as to accommodate with private apartments the noisy, and restless, and also the dying. In other respects it need not vary materially from ordinary hospital construction.

Although not strictly pertinent to the subject under discussion, it may not be unprofitable to briefly allude to the subject of buildings for the accommodation of the insane. In the brief time allotted to me, I can only direct your attention to the outlines of the subject, referring those who are interested in the details of asylum construction to the ample literature upon the subject, and especially to the excellent work of the late Dr. Kirkbride, of Philadelphia. The site for the construction of an asylum for the accommodation of the insane should be chosen—

1. With reference to its salubrity.

2. Its contiguity to an abundant and never-failing supply of good water.

3. With sufficient acreage of good soil to afford employment in its cultivation for forty per cent. of the expected maximum of the male population of the institution to be constructed. Other requisites, of minor character, need not be mentioned.

Buildings for the accommodation of the insane should be constructed—

1. With reference to the health of the inmates.

2. With reference to durability rather than ornament, though the attainment of the latter should not be wholly neglected.

3. The entire structure should be as near fire proof as possible. In the interest of economy, the main buildings should be three stories above the basement. The bedrooms of the patients should be large enough to allow one thousand cubic feet or space for each patient, exclusive of hall space.

Perhaps the most convenient general form for the construction of the buildings, is that of a crescent, with the concavity looking to the rear. In the center is placed the executive department, and residence apartments for the officers. In the rear, within the concavity formed by the two wings of the Asylum, is placed the boiler house, laundry, etc., at a safe distance from the main buildings in case of fire.

The basements under each wing should be mainly appropriated for common dining rooms, where all who are able to descend and ascend the stairways should take their meals. This arrangement would prove a measure of economy of space in the wards, as well as in the amount of food consumed.

Another advantage, would be the facility afforded for the inspection of the food by the officers of the Asylum.

Having thus briefly sketched the cardinal principles of the management of the insane, and also roughly outlined the necessary buildings for their accommodation, the writer, in concluding this paper, tenders to the Society his most sincere thanks for the patient hearing accorded him upon a subject not strictly medical, but nevertheless of the deepest interest to the medical profession, involving, as it does, the welfare of so large a proportion of the defective population of our country.

